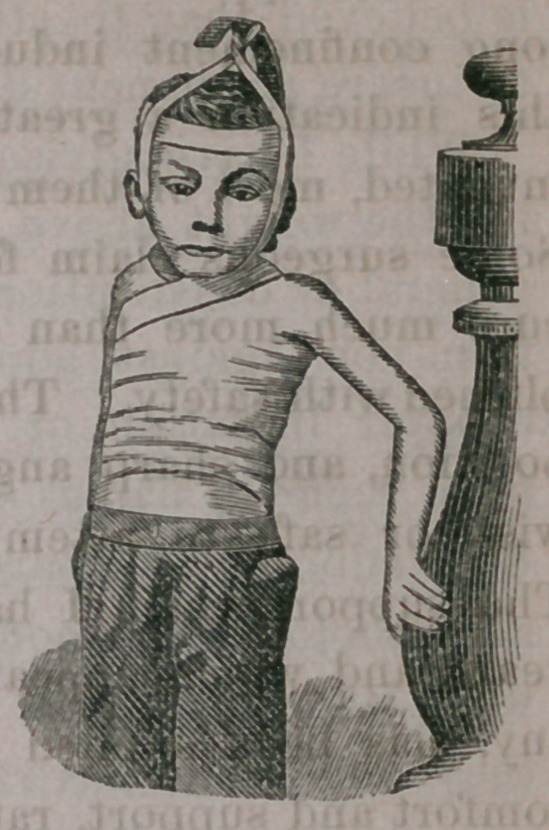# Clinical Remarks upon Surgical Cases in the Buffalo General Hospital—Radical Cure of Hydrocele—Spinal Curvature

**Published:** 1868-04

**Authors:** J. F. Miner


ART. II.—*Clinical Remarks upon Surgical Cases in the Buffalo General Hospital—Radical Cure of Hydrocele—Spinal Curvature*—By J. F. Miner, M. D.

*Gentlemen:*—We first present before you to-day, a case of hydrocele, and propose to make operation for what is called its "radical cure." The disease consists of an accumulation of serum in the cavity of the *tunica vaginalis testis*, or there is another form in which the accumulation takes place in a serous cyst of the spermatic cord; this is very rare, in comparison to its common occurrence in the vaginal tunic of the testicle. This disease may exist on both sides, or be only on one side; more commonly it is confined to one side; double hydrocele is said to be very infrequent in this country, so that this case which is double, has at least one feature thought to be of rare occurrence, and to be noticed on this account, though my own observation does not justify such belief. The history, appearance, feeling of fluctuation and transparency of the tumor when illuminated, as you now observe, by darkening the room and placing a light opposite, enable us to make diagnosis, and to distinguish hydrocele from the common forms of disease which in any degree resemble it. The fluid is not always clear and transparent; it may be bloody, milky, purulent, fibrinous, and in various other ways changed, so that this transparent appearance you observe may be absent in some instances. You must not allow this to lead you into error; blood is often mixed with the water when injury to the parts has been sustained, but the feeling of fluctuation and all the general symptoms of hydrocele are present, and will prove sufficient, in most cases, for correct diagnosis.

The effusion of serum into the cavity of the *tunica vaginalis testis*, it seems to me, must be the result of inflammation, even though no change in the condition or appearance of the membrane can be discovered. In some instances thickening, granulation, and other changes, obviously the effects of inflammation are plainly observable; and the presumption is, inflammation is present in all, though possibly of so low form that no change in the membrane secreting it, can be observed.

The questions of more especial clinical importance, are, how to distinguish it from diseases which resemble it, and how to operate for its cure. It resembles somewhat and has sometimes been mistaken for hernia, orchitis, sarcocele, malignant disease of the testicle, and, in rare instances, for some other forms of disease. Its history, peculiar shape, manner of growth, transparency, fluctuation, freedom from pain, together with absence of the symptoms which characterize other forms of disease with which it is liable to be confounded, will enable you to avoid error as to its true nature.

Various methods of cure have been proposed, such as inserting a seton through the cavity of the *tunica vaginalis testis*, injecting the cavity with tinct. iodine, solution of iodide of potass, port wine, or other stimulating fluid, or opening the cavity by long and free incision, and keeping the parts open in such manner as to expose the cavity to the stimulating influence of air until adhesive inflammation supervene. All operations yet proposed are open to objection, and nothing has yet been devised which is safe, certain, and wholly satisfactory. I inject the cavity from which the serum has been drawn with undiluted tinct. iodine, and this indicates sufficiently the plan which commends itself most strongly to my approval, while time will not permit the mention of the reasons for adopting it, or for excluding the others. You observe how the trocar and canula is introduced, and how injection of the iodine is made through the canula after the fluid has been withdrawn, thus avoiding all danger of throwing it elsewhere than desired. With this operation I have always succeeded, not always by the first effort, but always in the end, or after a second, or possibly in rare instances, a third trial.

Whatever plan of operation is adopted, the object is the same, viz: agglutination of the two surfaces of the serous membrane constituting the *tunica vaginalis testis*, thus obliterating this cavity, and uniting or closing in the surfaces which furnish the secretion.

*Spinal Curvature.*—We also present before you this morning an interesting case of angular curvature of the spine, or as sometimes called, posterior or tubercular curvature, which is a disease of very common occurrence, and which is of very great importance for you to understand. While I cannot enter into a discussion of spinal curvature; cannot even enumerate the various and conflicting opinions which have been entertained concerning its nature, causes or cure, I will try to point out briefly some of the more plain and obvious clinical facts concerning it. Since I cannot give you any idea of the various opinions of surgeons as to the nature or causes of this disease, much less any *resumé* of the arguments or modes of reasoning by which the various theories have been defended or sustained, it may be well to say simply, that the causes and true nature of spinal curvature is not fully determined—is not wholly understood. Many of the early views concerning it are no longer tenable, and the more recent and rational ones are not entirely satisfactory.

This little boy, now near eight years old, about one year since began to grow weak and pale, did not play and run with his usual activity, complained of being tired after slight exertion, lost his relish for food, became restless and disturbed in his sleep, and gave evidence that he was in some way unwell. Examination showed commencing deviation from the natural form of the spine. I was consulted at this early stage as to the nature and best remedy for his disease, but his parents being poor and his father dying soon after of acute disease, my advice could not be followed, and he has now been admitted to these wards for care which he did not receive at home. As yet there is no suppurative disease of the spinal bones or articular cartilages, but this is the same disease which does so often result in suppuration, and in some instances in formation of psoas abscess. Psoas abscess, then, is ulcerative disease of the bones, articular and connective tissues of the spine, and you will always examine the condition of the spine, when you estimate the importance, or attempt to treat what is called psoas abscess. In angular curvature of the spine you will also watch for symptoms of psoas abscess—for evidences of suppuration.

Protrusion of some one or more of the vertebral bones, tenderness upon percussion or pressure, deformity, and symptoms of general debility with considerable distress and discomfort, are the usual symptoms which accompany this disease. Many other and much more serious ones are sometimes added, especially in its advanced stages.

The indications for cure are to my mind quite obvious. The spine is no longer suited or capable of sustaining the weight of the head and upper part of the body, and this weight should be removed as far and as early as possible. The system should be sustained and never reduced by general or local depressing agencies. The bones, cartilages and ligaments of the spine are tender, irritable and painful, and all these conditions indicate relief from pressure, support and rest—rest if possible without confinement; long confinement induces debility and depression. To answer this indication a great many ingenious instruments have been invented, most of them more or less suited to the object in view. Some surgeons claim for their favorite machines and means of cure much more than can be accomplished—than can be accomplished with safety. The spine having once deviated greatly from position, and sharp angular curvature produced, it is not always wise or safe to attempt its restoration to perfect position.—The support which I have advised for these cases, for the past years, and which appears as satisfactory, safe, and successful as any, has been applied also to this case, and proves at least a comfort and support, rather than a torment and injury. It does not make great pretention to rectify all deviations, as is done by many others, but it is believed to answer all reasonable expectation.

You will allow me to quote from my former description of this instrument, and after five years' constant use, in a great variety of cases, confirm all that was then expressed in its favor. "Similar cases being frequent, an effort to supply mechanical support resulted in the invention and manufacture of an instrument, easily applied, comfortable and efficient, to which might be added a spring for sustaining the head, or this might be omitted in cases where the curvature was lower down the spine, or did not require that the head should be sustained. This was made of steel springs, fitting accurately to the spinal curve, and sustained at the base by a firm steel support passing around the pelvis, somewhat in the style of a truss. The following cuts represent the instrument and the spring appendage when applied, and by them a perfect idea of its construction may be gained:

1. Pelvic bands.
2. Abdominal elastic bands.
3—3. Shoulder straps.
4. Pad for pressure upon seat of curve.

This instrument can be made by mechanics who follow other trades; is stuffed so as to be comfortable, and even to afford relief to the youngest patients. It is made to fit accurately to the spine, and with it may be treated with some benefit all cases of spinal curvature, capable of mechanical relief. A pad is placed which may make pressure where required in posterior curve; lateral deviation is rectified by having an arm placed on either side as different cases may require, while an extending spring coming up over the head, will afford means of support in cases of curve or disease in the cervical bones, or in any cases where it is desirable to relieve the weight of the head. This instrument may be made useful, with its modifications and additions, in the treatment of a great variety of cases, and is to be preferred to more complicated machines in some respects; is more simple, less expensive, more comfortable to the patient, and for these reasons, if for no other, more likely to prove an available adjunct in the treatment of the various deformities of the spine.

Small children often suffer from this disease, and use of any retentive apparatus is regarded as uncomfortable, and with them, almost impossible of application; this is a mistake, it is not infrequent to obtain the greatest relief. *Extension* is often desirable, but *support* is useful; support which shall secure rest, without extending force, is oftentimes curative. As in fracture of bones, so in curvature of spine, rest will allow of cure; not, perhaps, without deformity, but, nevertheless cure, while in many cases, it is believed that to attempt perfect restoration of form, would be to insure permanent disease."

While I have advised means for relieving pressure—for sustaining the weight above the curvature, and have spoken of rest and general or constitutional support, I have almost forgotten to caution you against adopting that old and barbarous practice of counter irritation or derivation; blister, seton, issue and moxa, have been the names of the various modes of torture, which have been adopted as means of cure, in every way I believe opposed to all pathology and good reason. These means of cure were adopted when the tubercular theory of causation predominated, but how they could ever have been supposed capable of removing tubercular matter if deposited, or prevent or in any way overcome the tubercular tendency if present in the constitution, it is wholly impossible to conceive. Clinical experience was referred to for defense of this practice, but facts of this nature did not however exist except in the minds of those who had already determined to see the most favorable results, generally only in the minds of those who never saw unfavorable effects from favorite remedies.

Observe carefully for yourselves; meanwhile accept my assurance that these measures are not only wholly incapable of good but largely productive of evil, creating irritative fever, disturbing rest and appetite, and causing drain upon the system, now requiring support and rest. While I do not ask you to adopt my opinions, I beg of you to observe the effects of these remedies in the practice of others, as I am sorry to say, frequent opportunity will permit, before trying them in your own.

---

*Mortality after Amputation of the Thigh.*—M. Hussan, in publishing the statistics of the Hospitals of Paris, embracing the returns of 100,000 patients, gives the following results of amputation of the thigh: In 1861, 42 amputations, and only 7 recoveries; a mortality of 83.33 per cent. In 1862, 40 amputations, and 19 recoveries; a mortality of 52.50 per cent. In 1863, 40 rmputations, and 15 recoveries; a mortality of 62.50 per cent. Average mortality for three years, 66.11 per cent., or 41 recoveries in 122 operations.—*Boston Medical and Surgical Journal.*

## Figures and Tables

**Figure f1:**
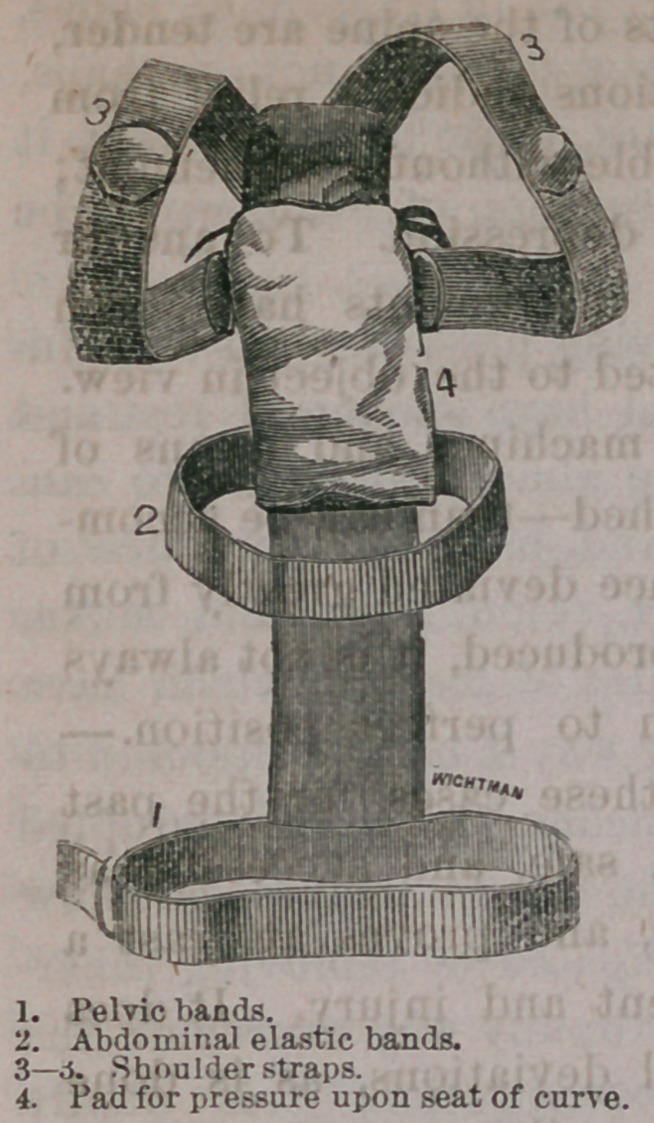


**Figure f2:**